# Long-term outcome of patients with active ankylosing spondylitis with etanercept-sustained efficacy and safety after seven years

**DOI:** 10.1186/ar4244

**Published:** 2013-06-20

**Authors:** Xenofon Baraliakos, Hildrun Haibel, Claudia Fritz, Joachim Listing, Frank Heldmann, Juergen Braun, Joachim Sieper

**Affiliations:** 1 Rheumazentrum Ruhrgebiet, Herne, Ruhr-University Bochum, Landgrafenstr. 15, 44652 Herne, Germany; 2 Department of Gastroenterology/Rheumatology, Charité, Medical University Berlin, Campus Benjamin Franklin, Hindenburgdamm 30, 12200 Berlin, Germany; 3 Epidemiology Department, German Rheumatism Research Center, Schumannstr. 21,10117 Berlin, Germany

**Keywords:** ankylosing spondylitis, TNFa, etanercept, ASDAS, BASDAI

## Abstract

**Introduction:**

Data from clinical studies on the long-term efficacy and safety of anti-tumor necrosis factor (TNF)-α therapy in patients with ankylosing spondylitis (AS) are scarce. This is the first report on continuous treatment with the TNFα fusion protein etanercept over seven years (y).

**Methods:**

Overall, 26 patients with active AS were initially treated with etanercept 2 × 25 mg s.c./week with no concomitant disease modifying anti-rheumatic drugs (DMARDs) or steroids. The clinical response was assessed by standardized parameters. The primary outcome was the proportion of patients in the Spondyloarthritis International Society (ASAS) partial remission at seven years. AS disease activity scores (ASDAS) for status and improvement were compared to conventional outcome measures.

**Results:**

Overall, 21/26 patients (81%) completed two years of treatment and 16/26 patients (62%) completed seven years. In the completer analysis, 31% patients were in ASAS partial remission at seven years, while 44% patients showed an ASDAS inactive disease status. Mean Bath AS activity index (BASDAI) scores, which were elevated at baseline (6.3 ± 0.9), showed constant improvement and remained low: 3.1 ± 2.5 at two years and 2.5 ± 2.2 at seven years, while ASDAS also improved (3.9 ± 0.7 at baseline, 1.8 ± 0.9 at two years, 1.6 ± 0.8 at seven years), all *P *<0.001. From the 10 dropouts, only 5 patients discontinued treatment due to adverse events. Patients who completed the study had lower baseline Bath AS function index (BASFI) scores vs. patients who discontinued. No other clinical parameter at baseline could predict any long-term outcome.

**Conclusions:**

This study confirms the clinical efficacy and safety of etanercept in patients with active AS over seven years of continuous treatment. After seven years, more than half of the initially treated patients remained on anti-TNF therapy, and one-third were in partial remission.

**Trial Registration:**

ClinicalTrials.gov: NCT01289743

## Introduction

Ankylosing spondylitis (AS), the prototype and the most severe form of the spondyloarthritides (SpA), is a frequent chronic inflammatory rheumatic disease with a prevalence of 0.1% to 1.1% [1]. AS affects young patients and frequently starts in the third decade of life, being initially characterized by inflammation of the axial skeleton but also by new bone formation in later stages. The socioeconomic burden of AS patients is substantial since absence from work and work disability are increased by three-fold [2-4].

Therapeutic options for patients suffering from AS have been limited over the last decades. There is no evidence that disease modifying anti-rheumatic drugs (DMARDs) work in axial manifestations of AS with the exception of sulfasalazine, which has some efficacy in AS patients with peripheral symptoms [5,6]. Non-steroidal anti-inflammatory drugs (NSAIDs) are considered first-line therapy in AS, while for patients with no response to NSAIDs treatment with anti-tumor necrosis factor (TNF)-α is the only alternative [7].

The efficacy of the recombinant 75 kD-TNF receptor IgG1 fusion protein etanercept has been demonstrated in clinical studies with patients with active AS [8-12], showing a significant decrease of disease activity already after the first two weeks [8-10] of treatment. In parallel, a significant decrease of inflammatory lesions in the sacroiliac joints and the spine as detected by magnetic resonance imaging (MRI) was detected as early as six weeks after the start of treatment [13,14].

After our first report from a placebo-controlled study of over three months with successful etanercept treatment in patients with initially active AS [8], and also successful and safe re-administration after treatment discontinuation (due to lack of medication at that time), we reported the clinical experience of the efficacy and safety of continuous therapy with etanercept over two years [15]. Similar data were reported also from other authors after two [16] or five years [17] of follow-up. This is the first report on clinical efficacy and safety of continuous treatment with etanercept over seven years in patients with established AS.

## Materials and methods

### Patients and study protocol

The local ethics committees of the universities of Berlin and Muenster approved the initial study [8] and the present extension, and patients gave written informed consent before participation. After the placebo-controlled phase of this trial [8] and treatment discontinuation, all patients (*n *= 26) entered the open-label phase of the study [18] if they had experienced a clinical relapse defined as a Bath AS disease activity index (BASDAI, [19]) ≥4 and a score of ≥4 on a 0 to 10 numeric rating scale (NRS) for spinal pain. All patients fulfilled the 1984 modified New York criteria [20] and were treated continuously with etanercept (2 × 25 mg s.c./week) for a total of seven years. Patients were allowed to continue on the same or a lower dose of NSAIDs during the study, as compared to their treatment before inclusion in the study.

### Assessment of clinical response

Disease status was assessed by using internationally accepted parameters for disease activity (ASDAS [21], BASDAI), function (BASFI, [22]) and metrology (BASMI, [23]). Furthermore, patient's (PatGA) and physician's (PhysGA) global assessments were also assessed on a 0 to 10 NRS. Laboratory parameters, such as C-reactive protein (CRP, mg/l), were measured by conventional means. The Short Form (SF)-36 questionnaire [24] was used to assess the health-related quality of life for both the physical and mental component using the algorithm of the Medical Outcome Trust [25].

Treatment efficacy was assessed by using the core set of criteria for symptomatic improvement in AS as suggested by the Assessment of SpondyloArthritis international Society (ASAS) [26], with a 20%, a 40% response, and an improvement in five out of the six criteria. These outcomes have been already described in detail [26,27]. Furthermore, ASAS partial remission (PR) was defined as a score ≤2 (on a NRS of 0 to 10) in each of the four ASAS 20% domains [26] and was compared with the definition of inactive disease status as defined in the ASDAS [21]. A status of low disease activity was defined as described in the ASDAS cut-offs [21] and was compared to a BASDAI ≤3, in accordance with a recent report by our group [28] and from previous data of the same study [15].

The primary endpoint of the study was the percentage of patients being in ASAS partial remission at Year 7. Similar data were collected for the ASDAS inactive disease status and these results were compared for each year of the study. Secondary endpoints were the proportion of patients who met the ASAS 40% criteria, '5-out-of-6' criteria as well as the mean improvement of the BASDAI, ASDAS, CRP, BASFI, BASMI and SF-36 after seven years.

### Statistical analysis

The analysis of the data was done as completer analysis. The Mann-Whitney-U test was used for comparison between subgroups of patients (for example, completers vs. dropouts) and the Wilcoxon test was used for comparison of variables between different time points. Fisher´s exact test was used for the comparison of categorical data. A significance level of 5% was used.

## Results

### Effectiveness of treatment

#### Baseline characteristics

Out of the 26 patients who were randomized into the original study [8], 21 patients (81%) completed the two-year and 16 patients (62%) completed the seven-year treatment period (14 males and 2 females, mean age at baseline 36.3 ± 7.5 years; range 23 to 52 years). The baseline disease characteristics of these patients did not differ from those who discontinued the study. Interestingly, in the BASFI status, the completers had a lower BASFI at baseline (5.3 ± 1.9) as compared to drop-outs (6.8 ± 1.6), but also this difference did not reach significance (*P *= 0.054). Table 1 shows the baseline characteristics of these two subgroups.

**Table 1:** Baseline disease characteristics of the patients

	Completer (*n *= 16)	Dropout (*n *= 10)
**age (years)**	36.3 ± 7.5	38.4 ± 11.0
**Male gender (n, %)**	14 (87.5%)	6 (60%)
**disease duration (years)**	13 ± 7.7	15 ± 10.8
**BASDAI (0 to 10)**	6.3 ± 0.9	7.0 ± 1.4
**BASFI (0 to 10)**	5.3 ± 1.9	6.8 ± 1.6
**BASMI (0 to 10)**	3.9 ± 2.2	3.7 ± 1.3
**CRP (mg/l)**	20.8 ± 17.7	19.3 ± 16.7
**ASDAS units**	3.9 ± 0.7	4.3 ± 0.9
**HLA positive (n patients, %)**	13 (81.3%)	10 (100%)

Baseline disease characteristics of the patients who completed Year 7 of the study (*n *= 16) and of those who discontinued during the seven years of treatment with etanercept. None of these parameters was significantly different when the two investigated groups were compared (all *P *>0.05)

#### Clinical efficacy of etanercept during the seven years of continuous treatment

**Figure 1:**
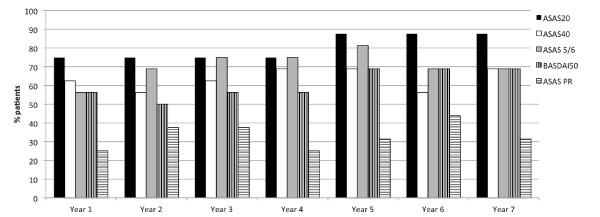
**Treatment outcome for each study year**. Response data for the 16 patients reaching Year 7. Treatment outcome according to established clinical parameters for each year of the study. ASAS20, ASAS 20% response, ASAS40, ASAS 40% response, ASAS 5/6, ASAS 5/6 response, BASDAI50, BASDAI 50% response, ASAS PR, ASAS partial remission. Shown are the response data for the 16 patients reaching Year 7 at different time points. ASAS, SpondyloArthritis international Society; BASDAI, Bath Ankylosing Spondylitis Activity Index

**Figure 2:**
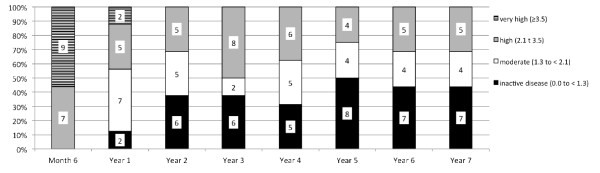
**Completer analysis for ASDAS status over seven years**. Completer analysis (*n *= 16) for the disease activity status as defined by the ASDAS over the entire study period of seven years. Numbers in the columns indicate the number of patients achieving the outcome. ASDAS, Ankylosing Spondylitis Disease Activity Score

**Figure 3:**
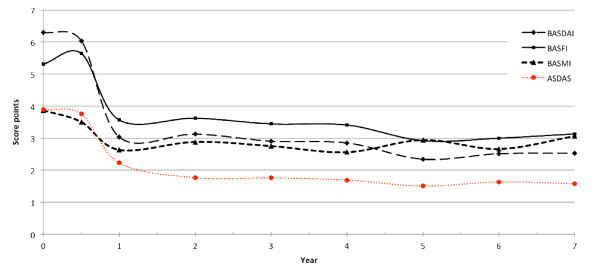
**Course of disease related clinical parameters: ASDAS, BASDAI, BASFI, BASMI**. Completer analysis. Course of disease related clinical parameters: ASDAS, Ankylosing spondylitis Disease Activity Score; BASDAI, Bath Ankylosing Spondylitis Disease Activity Index, BASFI, Bath Ankylosing Spondylitis Function Index; BASMI, Bath Ankylosing Spondylitis Metrology Index. Completer analysis of the 16 patients who reached Year 7 of the study.

Overall, 5/16 patients (31.3%) were in ASAS partial remission status (Figure 1), while 7/16 patients (43.8%) showed an ASDAS inactive disease status by the end of Year 7 (Figure 2). Mean ASDAS, BASDAI, BASFI and BASMI scores showed a rapid improvement, which remained stable over the entire study period (Figure 3). There was no change in mean values at Year 7 of the study as compared to the last report of the same patients after two years. The mean ASDAS decreased from 3.9 ± 0.7 units at baseline to 1.8 ± 0.9 units at two years and to 1.6 ± 0.8 units at seven years (both *P *<0.001 as compared to baseline), while the mean BASDAI decreased from 6.3 ± 0.9 at baseline to 2.5 ± 2.2 at seven years. A BASDAI of <3 units was achieved by 8/16 patients (50%) at Year 2 and by 11/16 patients (68.8%) at Year 7, while ASDAS moderate disease activity was achieved by 11/16 patients (68.8%) at both Year 2 and Year 7 (including those patients who were in ASDAS inactive disease, as described above).

**Figure 4:**
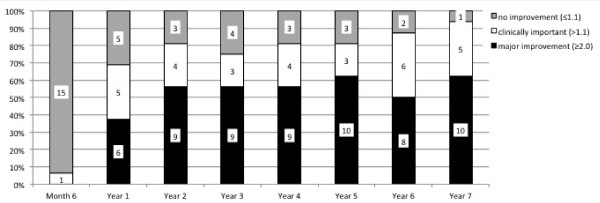
**ASDAS Change status for completers over seven years**. Change status of the ASDAS for the 16 completers over the entire study period of seven years. Numbers within the columns indicate the number of patients achieving the outcome. ASDAS, Ankylosing spondylitis Disease Activity Score.

The clinical improvement based on the ASAS criteria but also on the BASDAI-50% improvement indicated sustained benefit of treatment from the first year of the study and onwards up to Year 7 in any of the applied measures (Figure 1). ASAS40 response was achieved in 9/16 patients (56.3%) at Year 2 and in 11/16 patients (68.8%) at Year 7. An ASDAS major clinical improvement was achieved in 9/16 patients (56.3%) at Year 2 and in 10/16 patients (62.5%) at Year 7 (Figure 4).

#### Analysis of patients who did not show low disease activity status after seven years despite continuous treatment with etanercept

The analysis of the 4/16 patients (25%) who did not reach a BASDAI of <3 or ASDAS moderate disease activity at the end of Year 7 showed that 3 out of 4 of these patients had been in ASDAS high disease activity or ASDAS moderate disease at the end of each study year.

The BASDAI values for each of these four patients at the end of Year 7 ranged between 3.6 and 8.1, the ASDAS values ranged between 2.1 and 3.0 and the BASFI values ranged between 4.4 and 7.8. Furthermore, their CRP values improved during treatment and, although being increased prior to treatment, were normalized during the study period (mean: 4.2 mg/l, range 2.0 to 6.0 mg/l at the end of Year 7).

#### Changes in health-related quality of life (SF-36) and global assessments over seven years of etanercept treatment

Both components of the SF-36 improved significantly during treatment and remained at high levels until the end of the study. The PCS increased from 30.8 ± 6.8 points at baseline to 43.1 ± 7.7 points at Year 7, while the MCS increased from 40.6 ± 11.5 points at baseline to 46.7 ± 11.2 points at Year 7 (*P *<0.001 for the change in both components).

#### Correlations between ASDAS and other assessments of disease activity or treatment outcome

Cross tabulations were generated in order to evaluate the concordance of the categorical assessments based on ASDAS compared to the outcomes based on other measures (ASAS40, ASAS partial remission, low BASDAI status of <3 units).

The analysis of ASAS40 vs. ASDAS improvement (major, clinically important, or no improvement) showed that only one patient at years 2, 3 and 4 experienced an ASDAS major clinical improvement, but was regarded as an ASAS40 non-responder (Table 2).

**Table 2:** Cross tabulation of ASDAS change vs. ASAS40 response

	ASDAS change		
	
	major improvement	clinically important	no improvement
ASAS40			
**Year 1** Response Non-response	6 0	4 1	0 5

**Year 2** Response Non-response	8 1	1 3	0 3

**Year 3** Response Non-response	8 1	2 1	0 4

**Year 4** Response Non-response	8 1	3 1	0 3

**Year 5** Response Non-response	10 0	1 2	0 3

**Year 6** Response Non-response	8 0	1 5	0 2

**Year 7** Response Non-response	10 0	1 4	0 1

Numbers in the boxes represent the numbers of patients achieving the different combinations of responses.

Some more patients with discordant assessments were observed in the comparison of ASDAS status (inactive, moderate, high or very high disease activity) vs. ASAS partial remission, since the numbers of patients with ASDAS inactive disease in the absence of ASAS partial remission ranged between 1 and 4 at study years 2 to 7, while another patient had achieved ASAS partial remission in spite of high disease activity based on ASDAS at Year 7 (Table 3).

**Table 3:** Cross tabulation of ASDAS status vs. ASAS partial remission (PR)

	ASDAS status			
	
	Inactive disease activity	Moderate disease activity	High disease activity	Very high disease activity
ASAS partial				
**Year 1** Yes No	2 0	2 5	0 5	0 2

**Year 2** Yes No	4 2	2 3	0 5	0 0

**Year 3** Yes No	5 1	1 1	0 8	0 0

**Year 4** Yes No	3 2	1 4	0 6	0 0

**Year 5** Yes No	4 4	1 3	0 4	0 0

**Year 6** Yes No	6 1	1 3	0 5	0 0

**Year 7** Yes No	4 3	0 4	1 4	0 0

Numbers in the boxes represent the numbers of patients achieving the different combinations of responses.

Low BASDAI (<3 units) despite high ASDAS disease activity was noted in only very few patients, while one patient had no low BASDAI but was in ASDAS inactive disease at Year 2.

### Analysis of safety and reasons for treatment discontinuation over seven years

Episodes of recurrent uveitis were found in 7/16 patients (44%) during the entire study period, 5 of which developed uveitis during treatment with etanercept without a previous history of uveitis. In three of those patients, uveitis was noticed only once while the other two patients had 2 and 11 episodes of uveitis, respectively, during the seven years of the study. In the remaining two patients, who both had already reported uveitis at least once prior to inclusion in the study, there were one and six episodes of uveitis over seven years of treatment.

Overall, there were no differences in baseline status between those patients who completed the seventh year of the study and those who dropped out before Year 7 (data not shown). Ten patients of the initial 26 patients dropped out due to different reasons: the most common reason for discontinuation was moving to another place or lack of efficacy (both *n *= 2), while one patient discontinued because she wanted to become pregnant. The other reasons for discontinuation were heart discomfort, development of Crohn´s disease, reactivation of Crohn´s disease, lung carcinoma and death (probably due to heart attack), respectively. The two patients who discontinued due to lack of efficacy did not differ in their baseline characteristics from the others who discontinued due to an adverse event (data not shown).

## Discussion

This is the first report on seven years of treatment with the receptor fusion protein etanercept in patients with ankylosing spondylitis. Our data show that continuous treatment with etanercept over such a long time period was efficacious and safe.

All patients in this study were participants of the first placebo-controlled phase of the study and had already discontinued etanercept but had to resume therapy after about four months due to clinical relapse and a high level of disease activity. However, their clinical response after etanercept re-administration was similar to their status prior to discontinuation. These data have been reported elsewhere [8,28] and suggest that continuous treatment with TNF-blockers is recommended for the treatment of patients with active AS, as shown also with other anti-TNF compounds [28]. However, when needed, treatment discontinuation and then the resumption of therapy are possible without loss of clinical efficacy in the long term.

Importantly, the clinical efficacy of etanercept on disease activity, function and mobility was sustained over seven years. Both patient-oriented (BASDAI) and objective (CRP) measures for disease activity had already shown significant improvement at week 2 of the study [8], continued to improve during the first six months and remained on similar low levels over the entire study period, indicating no loss in the efficacy of etanercept after seven years. Furthermore, low BASDAI levels (a BASDAI of <3 units) and also ASDAS levels of <2.1 units were reported by the majority of the patients, indicating the sustainable effect of etanercept in the long-term treatment of active AS. Finally, the sustained improvements in the clinical composite measures or CRP were accompanied by stable improvements in the global assessment of the disease by patients and physicians, as well as in the mental and physical component scores of the SF-36.

Interestingly, not only parameters for disease activity but also function (BASFI) and metrology (BASMI) showed a similar course with sustained low levels over the seven years of the study. This is important information as compared to a very recent report on long-term anti-TNF treatment over eight years, where we had observed a slight increase of BASFI levels in comparison to previous years, a finding which also contributed to a slight decrease in the proportion of patients achieving partial remission at the end of that study. Although the number of patients in the current study is too limited to draw further conclusions, it seems that, overall, BASFI increases seem not to be a constant phenomenon in AS patients treated with anti-TNF for longer time periods. Furthermore, the reported restriction of mobility as measured by the BASMI remained at low levels over the seven years of etanercept treatment, supporting the efficacy of this compound in the long-term, especially with the known natural course of the disease and the expected continuous worsening of function and mobility in AS patients not treated with anti-TNF [29,30]. Overall, we found no correlation between the baseline clinical status of the patients and the response to or maintenance of treatment after seven years with a slight exception of the baseline BASFI status, which just reached statistical significance. In previous studies [31-33], younger age, lower BASFI, raised CRP or higher BASDAI were identified as predictors of a major (BASDAI50) clinical response in patients with active AS treated with TNF blockers. The results of the present study may be explained by the relatively low number of completers after seven years.

Another important result of this study is the comparison between established methods of disease activity status (BASDAI) or response to treatment (ASAS outcomes) and the recently introduced ASDAS method. Although ASDAS has been tested retrospectively in AS patients with a short-term follow-up, our study is, to our knowledge, the first report of ASDAS being tested as an outcome measure for a long-term follow-up. Both the BASDAI status but also ASAS outcomes were in good agreement with the ASDAS on status and on changing scores for disease activity during the long-term treatment with anti-TNF. The very few cases of discordance seemed to appear rather sporadically in single cases, thus indicating a high degree of concordance of the assessments made with the ASDAS compared to the outcomes based on the traditional disease activity measures. It is worth mentioning that in some patients this discordance was seen in ASDAS inactive disease compared to ASAS partial remission. This is an interesting result with respect to the long-term outcome aspect described herein. We believe that the reason for this observation has to do with impaired function that often occurs in the long-term course of the disease, even under effective anti-TNF blocker treatment. Since function is part of the ASAS partial remission criteria, this fact is most probably responsible for failure in reaching ASAS partial remission as a long-term outcome. It remains to be seen, in future long-term analyses and larger cohorts, whether reaching a low ASDAS early in the course of treatment can also be applied as a predictive measurement for long-term outcomes in AS patients treated with anti-TNF, something which has already been shown for a low BASDAI [34].

Of note, four patients remained in the study all seven years despite showing a relatively high disease activity score. All these patients wished to continue treatment with etanercept and in these patients a clear reduction of their CRP values occurred which, interestingly, were also even lower than the CRP values of the other 12 patients, with the exception of year 5. More long-term studies would be necessary to investigate the importance of a constantly low CRP value on the long-term outcome of AS.

The long-term safety outcome in the present study was very good. Overall, >60% of the initially included patients remained on continuous treatment with etanercept over seven years. This is the highest rate of drug survival over such a long-term treatment period reported in the literature so far for any patients treated with anti-TNF. Even in studies with AS patients, who overall reported higher rates of drug survival as compared to studies with rheumatoid arthritis [35-37], drop-out rates were reported to be as high as 50% of the initially included patients [34]. However, as recently reported [34], a substantial proportion of patients may discontinue their participation in a clinical study in the long-term also due to other reasons than adverse events. We did not see any specific signs of safety issues in the patients who discontinued etanercept treatment in our study. However, it is important to mention that we did see newly developed Crohn´s disease in one of our patients as well as the reactivation of Crohn´s disease in another patient under etanercept treatment. The potential of reactivation of Crohn´s disease under treatment with etanercept has already been described in an extensive meta-analysis some years ago [38]. Nevertheless, at present, there is no evidence that etanercept leads more often to flares of Crohn´s disease or any other inflammatory bowel diseases as compared to patients with AS who are not being treated with anti-TNF [38]. Another important safety aspect that needs to be mentioned is the recurrent uveitis rates in a substantial number (44%) of patients, as well as the fact that the majority of those patients developed uveitis after treatment initiation. Nevertheless, the majority of them (71%) showed no more than 2 episodes during the seven years of the study, while, on the other hand, one patient reported 11 episodes during the entire study period. In a recent analysis of several etanercept trials in AS, the uveitis rates were lower in these patients before treatment with etanercept and were lower compared to patients treated with a placebo [39].

## Conclusions

In conclusion, in this long-term follow-up study with AS patients treated continuously over seven years, etanercept showed a sustained efficacy and good safety profile, confirming the usage of TNF-blockers in patients with active disease. Drug survival rates were high in this small cohort. The ASDAS is reliably assessing the long-term outcome of AS patients treated with anti-TNF. Its use as a predictive tool for long-term treatment outcome needs to be shown in larger studies.

## Abbreviations

AS: Ankylosing spondylitis; ASAS: SpondyloArthritis international Society; ASDAS: Ankylosing spondylitis disease activity score; BASDAI: Bath ankylosing spondylitis activity index; BASFI: Bath ankylosing spondylitis function index; BASMI: Bath ankylosing spondylitis metrology index; CRP: C-reactive protein; DMARDs: Disease modifying anti-rheumatic drugs; MRI: Magnetic resonance imaging; NRS: Numeric rating scale; PatGA: Patient´s global assessment; PhysGA: Physician´s global assessment; PR: Partial remission; SF-36: Short-form 36 questionnaire; SpA: Spondyloarthritides; TNF: Tumor necrosis factor

## Competing interests

XB, HH, FH, JB and JS have received research grants, consultant fees and advisory board and speaker´s honoraria from Abbott, Celgene, Centocor, Chugai, MSD, Novartis, Pfizer and UCB. CF and JL have no competing interests.

## Authors' contributions

XB contributed to the organization of the study, clinical visits with patients and wrote the manuscript. HH contributed to the organization of the study, clinical visits with patients, and editing of the manuscript. CF and JL were responsible for analyzing statistics, and editing the manuscript. FH contributed to clinical visits of patients and the editing of the manuscript. JB and JS conceived the idea for the study, were responsible for the organization of the study, and contributed in the writing of the manuscript. All authors read and approved the final manuscript.
